# Short-Term Effects of Exposure to Atmospheric Ozone on the Nasal and Respiratory Symptoms in Adolescents

**DOI:** 10.3390/toxics13030196

**Published:** 2025-03-09

**Authors:** Yoshiko Yoda, Takeshi Ito, Junko Wakamatsu, Tomonari Masuzaki, Masayuki Shima

**Affiliations:** 1 Department of Public Health, School of Medicine, Hyogo Medical University, Nishinomiya 663-8501, Hyogo, Japan; yoda@tamateyama.ac.jp; 2 Faculty of Health Sciences for Welfare, Kansai University of Welfare Sciences, Kashiwara 582-0026, Osaka, Japan; 3 National Institute of Technology, Yuge College, Kamijima 794-2593, Ehime, Japan; t.ito@yuge.ac.jp (T.I.); wakamatsu@yuge.ac.jp (J.W.); t_masuzaki@yuge.ac.jp (T.M.); 4 School of Nursing, Hyogo Medical University, Kobe 650-8530, Hyogo, Japan

**Keywords:** atmospheric ozone, indoor ozone, personal exposure, nasal symptoms, respiratory symptoms

## Abstract

Information regarding the effects of exposure to relatively low ozone (O_3_) concentrations in daily life is limited. We evaluated the effects of daily O_3_ exposure on nasal and respiratory symptoms in healthy students. A panel study was conducted with students (39 people) for approximately one month. They were asked to record the presence or absence of any nasal or respiratory symptoms each day. O_3_ concentrations were continuously measured inside and outside the classrooms, and the maximum 1 h, maximum 8 h average, and 24 h average values were calculated for the 24 h before recording the symptoms. Additionally, personal exposure to O_3_ was repeatedly measured every 24 h using passive samplers. Mixed-effects models were used to evaluate the association between daily symptoms and various O_3_ concentrations. Increases in maximum 1 h concentrations of indoor and outdoor O_3_ were significantly associated with the occurrence of nasal congestion and runny nose, respectively. These associations were more pronounced in those with a history of pollinosis or allergic rhinitis. Personal O_3_ exposure per 24 h was also associated with sneezing, runny nose, and nasal congestion. This study showed that the assessment of the amount of personal exposure levels is desired to evaluate the health effects of O_3_ exposure.

## 1. Introduction

Photochemical oxidant (Ox) concentrations are on the rise globally. In 2020, the United States Environmental Protection Agency (US EPA) assessed that Ox comprises various health effects, including on the respiratory system [1], making it a serious international issue. In Japan, the environmental standard for Ox is set at 0.06 ppm or less for 1 h average concentrations. However, almost none of the monitoring stations across the country recorded these target values [2]. Photochemical oxidants are secondary air pollutants produced by exposure to ultraviolet light from atmospheric nitrogen oxides and volatile organic compounds (VOCs), and the majority constitute ozone (O_3_) [3]. The Global Burden of Disease Study 2017 [4] reported that the estimated number of deaths from chronic obstructive pulmonary disease due to atmospheric O_3_ increased by 20.4% from 2007 to 2017. The World Health Organization (WHO) [5] updated its air quality guidelines in 2021, setting the guideline values for ambient air O_3_ concentrations as a maximum 8 h average of 100 μg/m^3^ or less and a 6-month moving average of 60 μg/m^3^ or less during the peak season.

Many studies have demonstrated the effects of O_3_ on human health. A meta-analysis in China showed an association between ambient O_3_ exposure and a significant increase in mortality from respiratory diseases [6]. Global estimates posit that deaths from respiratory diseases will increase by more than one million per year [7]. Epidemiological studies showed that exposure to relatively high O_3_ concentrations can affect the respiratory system [8,9,10,11]. Studies indicated that exposure to O_3_ is associated not only with asthma but also with symptoms of allergic rhinitis and pollinosis [12,13]. However, currently most studies have evaluated the relationship between outdoor O_3_ concentrations and health effects. Therefore, much remains unknown regarding the effects of O_3_ exposure in daily life environments on human health. As most people tend to spend longer time indoors than outdoors, it is necessary to consider indoor O_3_ concentrations [14,15]. Studies evaluating indoor O_3_ concentrations and personal exposure reported that even low O_3_ concentrations can affect the respiratory system [16,17].

A panel study in Greece reported that increases in the daily maximum 8 h average of O_3_ were associated with increased nasal congestion and coughing among school children [18]. Airborne O_3_ concentrations in offices in eight European countries were associated with nasal and respiratory symptoms among employees [19]. Associations were also reported between short-term exposure to O_3_ and coughing, nasal congestion, and lung function [20]. A study in human volunteers reported that exposure of healthy adults to 0.06 ppm O_3_ for 6.6 h increased neutrophil inflammation in the airways 18 h later [21]. In animal studies, mice exposed to O_3_ developed acute neutrophilic rhinitis, exhibiting airway epithelial necrosis and overgrowth of mucosal cells [22]. The mechanism by which O_3_ exposure causes rhinitis in rats was shown to be due to the fact that inhalation of O_3_ increases Th2 cytokines and reduces interferon-gamma (IFN-γ), which is a protein regulating inflammatory and immune responses. This process disrupts the redox balance in the airways and causes oxidative damage to the airways [23,24].

O_3_ concentrations are not constant throughout and tend to be higher during the daytime. Earlier studies examined the relationships between various O_3_ indicators and health effects. Panel studies in China found that the maximum 8 h average daily value was associated with decreased pulmonary function and abnormal electrocardiograms [25,26]. Reportedly, the maximum 1 h value and the maximum 8 h average value for daily mortality are more strongly associated with daily mortality than that with the 24 h average value [27], suggesting that high O_3_ concentrations throughout the day would likely have negative effects on health. In Japan, the environmental standard has been set for 1 h values of O_3_. Simultaneously, the WHO and US EPA also set environmental standards, stating that the maximum 8 h average daily value is the most relevant to health [1,5].

An increase in atmospheric O_3_ concentrations from spring to summer is observed in Japan. Particularly, the Seto Inland Sea region is uniquely surrounded by mountains and the sea and is known as an area with high atmospheric O_3_ concentrations [11]. We aimed to evaluate the O_3_ concentrations to which healthy adolescent students are exposed daily and to clarify their effects on daily nasal and respiratory symptoms. Additionally, we examined whether the effects differed depending on whether the individual had a history of pollinosis or allergic rhinitis.

## 2. Materials and Methods

### 2.1. Study Design and Population

This study was conducted on Yuge Island, located almost in the center of the Seto Inland Sea in western Japan. This place has a unique topography, with remote islands dotted across the sea surrounded by mountains. The Seto Inland Sea is an area with heavy traffic by many vessels, including fishing boats and cargo ships [11]. Yuge Island is a remote island with an area of 8.61 km^2^. There are no major anthropogenic emission sources such as factories and busy roads on the island. A survey of 39 healthy students attending a technical college on the island (aged 16–17 years, 24 males and 15 females) was conducted to investigate their daily nasal and respiratory symptoms while measuring O_3_ concentrations both inside and outside the school. The survey period was approximately one month, from 10 May to 7 June 2022, when O_3_ concentrations are likely to be high without long holidays or special events in the school.

This study was approved by the Ethics Committee of Hyogo Medical University (No. 3988; 22 February 2022).

### 2.2. Exposure Assessment

O_3_ concentrations were continuously measured using ultraviolet absorption ozone monitors (ELM-1, Ebara Jitsugyo Co., Ltd., Tokyo, Japan) installed in classrooms in the target schools where students usually spent a lot of time. Additionally, on the rationale that indoor O_3_ concentrations are caused by outdoor inflow [28], measuring devices were also installed on the classroom balconies. O_3_ concentrations were recorded at 1 min intervals, and hourly averages were calculated. To analyze the relationship with health effects, the maximum 1 h value, the maximum 8 h average, and the 24 h average from 8:00 a.m. the previous day to 8:00 a.m. the following day were calculated. Furthermore, 23 students (13 males and 10 females), who consented to measure their personal O_3_ exposure during the study period, carried a small passive sampler (OG-SN-S, Ogawa & Co., Ltd., Kobe, Japan) equipped with an O_3_ collection filter, with the filter being replaced every morning at 8:00 a.m. at 24 h intervals. Over the weekend, samples were collected for 72 h at mornings from Friday to Monday, and the collected samples were analyzed using ion chromatography (Basic IC plus, Metrohm AG, Herisau, Switzerland). Temperature and relative humidity were also measured inside and outside the classroom during the study period using a HOBO data logger (Onset Computer Corporation, Bourne, MA, USA). The concentrations of particulate matter ≤ 2.5 μm in diameter (PM_2.5_) were continuously measured using an automatic dichotomous β gauge monitor (SPM-613D, Kimoto Electric Co., Ltd., Osaka, Japan) on the rooftop of the school. Because the concentrations of nitrogen dioxide (NO_2_) were not measured in the island, the data were collected from the nearest monitoring station in another island in the Seto Inland Sea (approximately 20 km).

### 2.3. Measures of Health Outcomes

Before the start of the study, a standard questionnaire [29] was conducted to assess the presence or absence of a history of pollinosis or allergic rhinitis. During the study period, the participants were asked to fill out a daily symptom survey using Google Forms at 8:00 a.m. every morning on whether they had nasal or respiratory symptoms, such as sneezing, runny nose, stuffy nose, coughing, and shortness of breath.

### 2.4. Statistical Analysis

An analysis was performed using a mixed-effects model to assess the relationship between the presence or absence of daily nasal and respiratory symptoms and O_3_ concentrations. This model is appropriate for evaluating repeated surveys [30]. Confounding factors considered were gender, history of pollinosis or allergic rhinitis, PM_2.5_ concentrations, temperature, and relative humidity. Stratified analyses were also conducted depending on the presence or absence of a history of pollinosis or allergic rhinitis. For subjects who underwent personal O_3_ exposure measurements, the relationships between the presence or absence of daily nasal symptoms, personal O_3_ exposure, and the 24 h average O_3_ concentration inside and outside the classroom were analyzed. Given the small number of symptomatic individuals, respiratory symptoms were excluded from the analysis of the associations with individual exposure. The results were expressed as odds ratios (ORs) and 95% confidence intervals (CIs) for daily symptom occurrence per interquartile range (IQR) increase in each O_3_ concentration. In addition, we performed two sensitive analyses. First, NO_2_ was added as a covariate to estimate the potential confounding effect of co-pollutant. Second, the analysis was restricted to the subjects without missing records during the study period (*n* = 31).

All analyses were performed using SPSS 29 (IBM Co., Armonk, NY, USA), with *p* < 0.05 considered statistically significant.

## 3. Results

### 3.1. Descriptive Statistics

Table 1 shows the characteristics of the subjects. A total of 24 men and 15 women were present, with a mean age of 16.1 ± 0.3 years. Overall, 25 (64.1%) had a history of pollinosis or allergic rhinitis. A total of 1117 people responded to questions about their daily symptoms during the study period, and the overall prevalence rate was the highest for runny nose at 11.4%, followed by nasal congestion at 8.0% and sneezing at 7.6%. The rates of cough and dyspnea were low at 2.6% and 1.9%, respectively.

Table 2 shows the outdoor and indoor O_3_ concentrations, temperature, and relative humidity during the study period. The maximum 1 h values of outdoor and indoor O_3_ concentrations were 141.5 ± 57.2 ppb and 98.6 ± 45.7 ppb, respectively. The maximum 8 h average values were 83.0 ± 36.7 ppb and 65.0 ± 31.1 ppb, respectively, and the 24 h average values were 45.4 ± 20.1 ppb and 44.4 ± 20.4 ppb, respectively. In all parameters, the O_3_ concentrations outdoors were higher than those of the indoors, although the indoor concentrations were also relatively high because the windows were left open for long periods during the day. The concentrations of PM_2.5_ and NO_2_ were considerably low throughout the study period.

### 3.2. Relationship Between Indoor and Outdoor O_3_ Concentrations and Daily Symptoms

**Figure 1:**
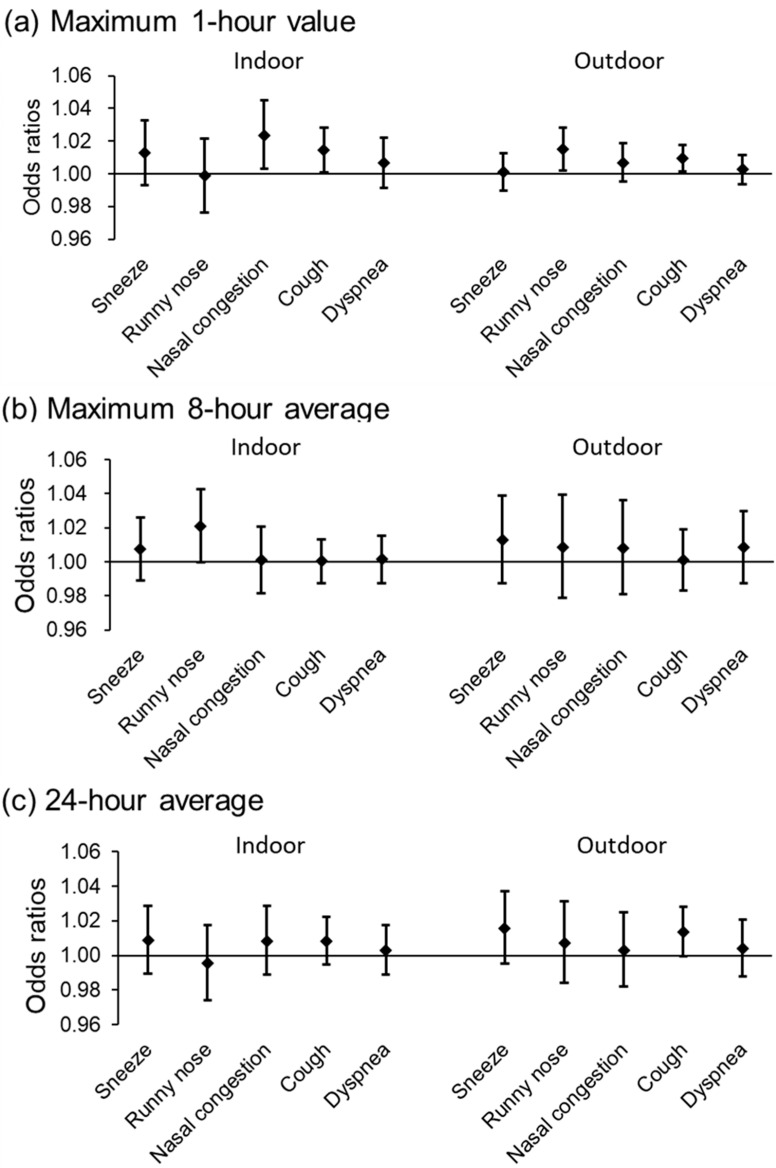
Association of various indoor and outdoor ozone concentrations with daily nasal and respiratory symptoms: (**a**) Maximum 1 h value; (**b**) Maximum 8 h average; (**c**) 24 h average.

Figure 1 shows the relationship between indoor and outdoor O_3_ concentrations and daily nasal and respiratory symptoms. An increase in the maximum 1 h value indoor O_3_ significantly associated with the occurrence of nasal congestion (OR: 1.02 [95% CI: 1.00–1.05] per IQR increase (80.4 ppb)). The maximum 1 h value of outdoor O_3_ was significantly associated with runny nose (OR: 1.02 [95% CI: 1.00–1.03] per IQR increase (70.4 ppb)) and cough (OR: 1.01 [95% CI: 1.00–1.02]. The associations between the maximum 1 h values of indoor and outdoor O_3_ and sneezing and dyspnea were insignificant. Runny nose was also significantly with the maximum 8 h average of indoor O_3_. In addition, no significant association was observed between 24 h average values of indoor and outdoor O_3_ concentrations and nasal and respiratory symptoms.

**Figure 2:**
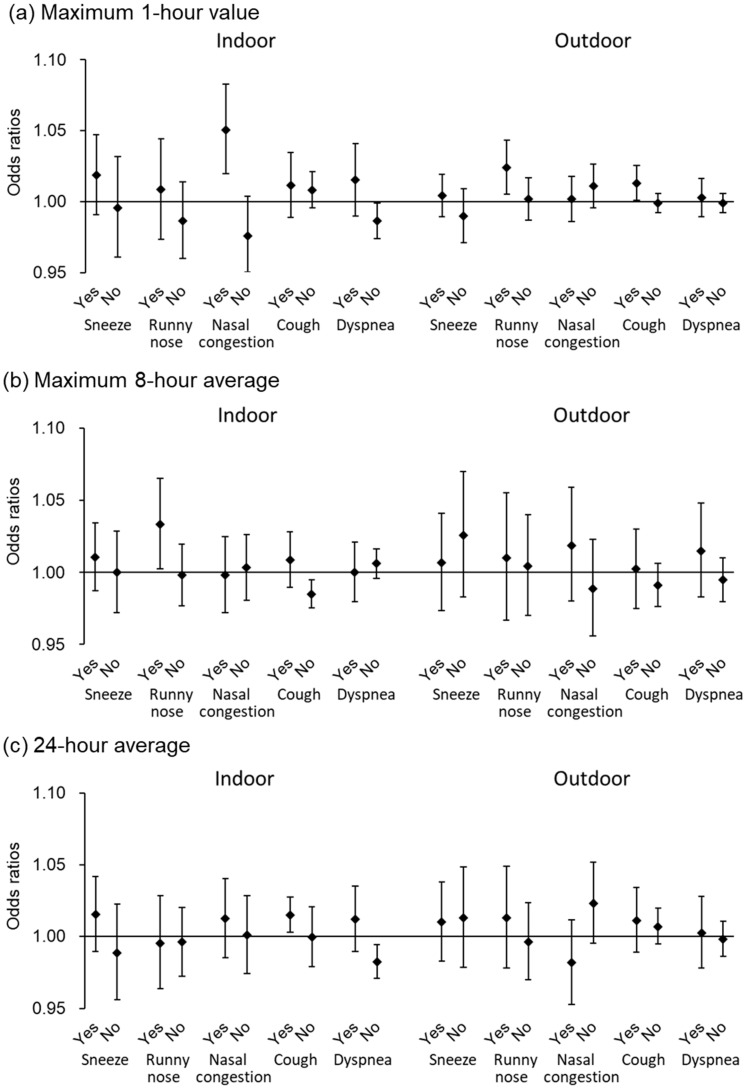
Association of various indoor and outdoor ozone concentrations with daily nasal and respiratory symptoms, by a history of pollinosis and/or allergic rhinitis: (**a**) Maximum 1 h value; (**b**) Maximum 8 h average; (**c**) 24 h average.

Figure 2 shows the results depending on the presence or absence of a history of pollinosis or allergic rhinitis. Among those with a history, an increase in the maximum 1 h value of indoor O_3_ was associated with a significant increase in nasal congestion (OR: 1.05 [95% CI: 1.02–1.08]), and an increase in the maximum 1 h value of outdoor O_3_ was associated with a significant rise in runny nose (OR: 1.02 [95% CI: 1.01–1.04]) and cough (OR: 1.01 [95% CI: 1.00–1.03]). Furthermore, the maximum 8 h average indoor value was significantly associated with runny nose (OR: 1.03 [95% CI: 1.00–1.07]). The 24 h average of indoor O_3_ was associated with cough (OR 1.02 [95% CI: 1.00–1.03]). However, the maximum 8 h average and the 24 h averages of outdoor O_3_ were not significantly associated with any symptoms. Those without a history of pollinosis or allergic rhinitis exhibited no association between any O_3_ concentrations and symptoms. No significant association was observed between PM_2.5_ concentrations and any nasal and respiratory symptoms.

We also investigated potential confounding from ambient NO_2_ concentrations. Significant associations between indoor and outdoor O_3_ concentrations and nasal and respiratory symptoms remained unchanged after adjusting for NO_2_ (Table S1). In the analyses restricted for the subjects without missing records during the study period, the results were generally robust (Table S2).

### 3.3. Relationship Between Personal O_3_ Exposure and Daily Symptoms

**Figure 3:**
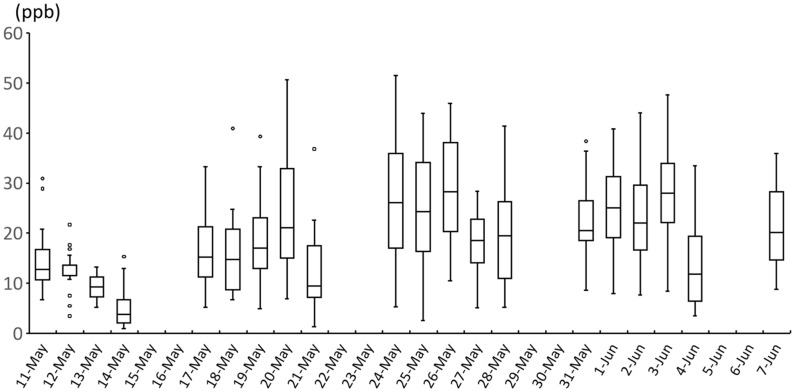
Trend of personal exposure levels to ozone during the study period.

**Figure 4:**
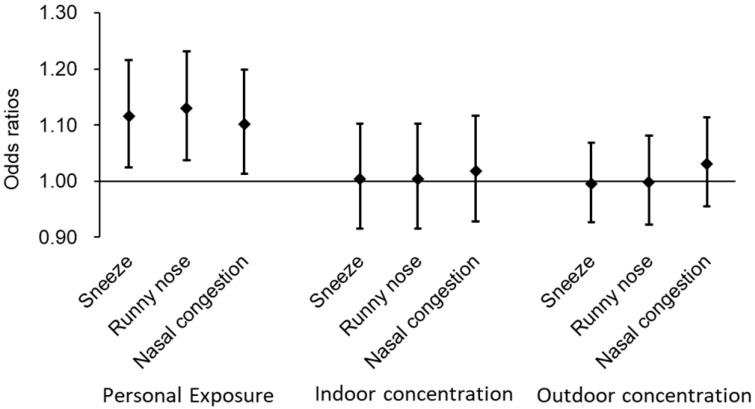
Association of personal exposure, indoor and outdoor ozone concentrations with daily nasal symptoms.

The valid measurements of personal O_3_ exposure were obtained from 23 students throughout the study period. The characteristics of the subjects who measured personal O_3_ exposure were similar to those of subjects who did not measure (Table S3). The mean of personal O_3_ exposure ranged from 8.2 to 36.0 ppb, depending on the subject. The overall mean was 18.8 ± 6.4 ppb, which is significantly lower than the indoor and outdoor O_3_ concentrations during the same period. The changes in daily personal exposure are shown in Figure 3. Figure 4 shows the relationship between 24 h personal O_3_ exposure and 24 h average indoor and outdoor exposure levels and nasal symptoms. As personal O_3_ exposure increased, sneezing, runny nose, and nasal congestion all significantly increased (OR: 1.12 [95% CI: 1.02–1.22], 1.13 [95% CI: 1.04–1.23], and 1.10 [95% CI: 1.01–1.20] per IQR increase (14.8 ppb), respectively). Even when limited to the subjects for whom personal exposure was measured, the 24 h average indoor and outdoor O_3_ during the same period was not significantly associated with any symptoms.

## 4. Discussion

This study aimed to investigate the effects of atmospheric O_3_ exposure on nasal or respiratory symptoms among healthy adolescent students in daily life. We analyzed the association between the presence or absence of and three indicators of O_3_: the maximum 1 h value, the maximum 8 h average value, and the 24 h average value. As a result, a significant association was found between the maximum 1 h values indoors and outdoors and runny nose, stuffy nose, and coughing. However, no significant correlation existed between the maximum 8 h average values or the 24 h average values and any of the symptoms. The subjects of our study were adolescent students. We surmise that O_3_ exposure caused an inflammatory reaction in their noses and airways, resulting in nasal and respiratory symptoms. Thus, the indoor and outdoor O_3_ concentrations were associated with nasal and respiratory symptoms, with a strong association being observed with the maximum 1 h value. This may be due to the reasonably high maximum 1 h values both indoors and outdoors. Earlier panel studies in European countries indicate that short-term O_3_ exposure affects the nose and respiratory tract [18,19,20], which are consistent with that of the results of our study.

The association between the maximum 1 h values of indoor and outdoor O_3_ and nasal and respiratory symptoms was stronger in the subjects with a history of pollinosis or allergic diseases than that in people without it. The association between the maximum 8 h average of indoor O_3_ and runny nose was also significant. We previously reported that people with a history of asthma experienced a decline in lung function scores in response to increased indoor O_3_ concentrations. However, O_3_ induced no changes in lung function scores in people with a history of allergies [11]. Contrarily, Niu et al. reported that airway inflammation due to O_3_ exposure was more pronounced in individuals with allergic diseases than in healthy individuals [31]. Thus, no consensus was reached regarding the difference in the effects of O_3_ exposure depending on whether or not one has a history of allergic disease. This may be attributed to differences in study design and subject characteristics. In this study, we evaluated the association with nasal and respiratory symptoms and found that individuals with a history of allergic disease were more susceptible to O_3_ exposure effects.

Currently, most epidemiological studies have used outdoor O_3_ concentrations as an exposure index. However, given that people tend to spend longer time indoors, indoor and personal exposure concentrations need to be considered to correctly evaluate the health effects of O_3_ [15]. A report indicated that exposure to indoor O_3_ during sleep, even at low concentrations, can affect the respiratory system of young people [17]. A panel study in Shanghai found that daytime personal O_3_ exposure was only about half the atmospheric air concentration but was strongly associated with airway inflammation [31]. In Greece, weekly personal O_3_ exposure in children, measured using personal samplers, was significantly lower than that of the outdoor concentrations but was found to be associated with airway inflammation and respiratory symptoms [16]. In this study, personal exposure for some subjects was measured repeatedly every 24 h using personal samplers to examine the relationship with daily symptoms. The results showed that personal O_3_ exposure was significantly associated with all the following symptoms: sneezing, runny nose, and nasal congestion. Although the 24 h average indoor and outdoor O_3_ concentrations were approximately twice that of the individual exposure levels, no association was found with any symptoms. This result is consistent with that of earlier studies and indicates that measuring personal exposure is desirable for assessing the health effects of O_3_. The effects of PM_2.5_ on any nasal and respiratory symptoms were not observed. Additionally, our previous studies in the region found no respiratory effects of particulate matter in the spring [32]. Additionally, the effects of NO_2_ were also observed. No major artificial sources of air pollution exist around the remote island where this study was conducted, and the concentrations of PM_2.5_ and NO_2_ were considerably low during the study period [11].

One strength of this study is that to evaluate O_3_ exposure in daily life, we continuously measured O_3_ concentrations both indoors and outdoors in rooms where the subjects spent a long time. We used the maximum 1 h value, maximum 8 h average, and 24 h average values in the 24 h before recording daily symptoms. Personal O_3_ exposure was also measured repeatedly every 24 h to assess the associations with daily symptoms. The results showed that the association between nasal and respiratory symptoms differed depending on the exposure indicator to O_3_. We believe that these findings will be instrumental in evaluating the short-term effects of O_3_ exposure correctly and in preventing health effects.

This study has some limitations. First, the subject size was small because they were limited to students in the classes where O_3_ concentrations were measured. The subjects were healthy students on a remote island without major anthropogenic emission sources, and the number of students was limited. In particular, the subjects for the analysis of personal exposure were only 23 students. Although all students in the class were asked to participate in the measurement of personal O_3_ exposure, some students did not consent or dropped out from filter replacement every morning during the study period. However, by recording symptoms daily for approximately one month, we could examine the relationship between the responses of a total of 1117 and 656 records and daily O_3_ concentrations and personal O_3_ exposure, respectively. Second, the amount of pollen in the air, thought to affect nasal symptoms [12], was not considered. Reportedly, high O_3_ concentrations increase the impact of pollen exposure on the airways [33]. In Japan, the amount of cedar and cypress pollen, which are reported to be major allergens of pollinosis, is high from February to April but low from May to June, when this study was conducted [34]. Furthermore, there were no cedar and cypress plantations around the island. Third, this study was only conducted for one month, from May to June. A period when O_3_ concentrations were relatively high with no school long holidays or special events was selected to allow for daily survey cooperation. In order to generalize the findings of our study, participants of all ages should be recruited and various health outcomes should be measured across several whole years in future studies.

## 5. Conclusions

Short-term O_3_ exposure in daily life was associated with nasal and respiratory symptoms in students, especially those with a history of pollinosis or allergic rhinitis. The association with the maximum 1 h values was among the three time-unit concentrations. An association between 24 h average personal exposure and nasal symptoms was also observed. To evaluate the health effects of O_3_ exposure, an assessment of the amount of personal exposure levels are preferable.

## Figures and Tables

**Table 1:** Characteristics of study subjects.

	Male (*n* = 24)	Female (*n* = 15)	Total (*n* = 39)
Age (years), mean (SD)	16.1 (0.3)	16.1 (0.3)	16.1 (0.3)
History of pollinosis and/or allergic rhinitis, n (%)	17 (70.8)	8 (53.3)	25 (64.1)
Response to questionnaire on daily symptoms	*n* = 683	*n* = 434	*n* = 1117
Sneeze (%)	7.0	8.5	7.6
Runny nose (%)	11.6	11.1	11.4
Nasal congestion (%)	7.6	8.5	8.0
Cough (%)	2.5	2.8	2.6
Dyspnea (%)	2.6	0.7	1.9

SD, standard deviation.

**Table 2:** Distribution of daily levels of environmental variables during the study period.

	Mean	SD	Percentile					IQR
			Minimum	25th	Median	75th	Maximum	
Outdoor								
O_3_ 1 h maximum, ppb	141.5	57.2	49.4	107.4	141.6	177.8	290.4	70.4
O_3_ Maximum 8 h average, ppb	82.2	37.0	7.3	56.9	76.4	112.4	152.8	55.6
O_3_ 24 h average, ppb	45.4	20.1	3.1	33.3	46.2	62.6	75.0	29.3
Temperature, °C	22.6	1.9	18.4	21.0	22.4	24.4	26.0	3.4
Relative humidity, %	57.6	10.0	44.2	50.4	54.1	62.5	80.2	12.1
PM_2.5_ 24 h average, μg/m^3^	9.1	3.5	3.2	7.0	8.7	10.8	18.9	3.8
NO_2_ 24 h average, ppb	4.3	1.9	1.3	3.1	3.7	5.0	8.2	1.9
Indoor								
O_3_ 1 h maximum, ppb	98.6	45.7	24.6	60.7	88.7	141.1	197.2	80.4
O_3_ Maximum 8 h average, ppb	66.8	30.1	12.4	42.2	73.5	84.4	128.4	42.2
O_3_ 24 h average, ppb	44.4	20.4	9.5	27.5	44.0	56.8	89.7	29.3
Temperature, °C	26.3	2.3	23.0	24.1	26.1	28.1	30.1	4.0
Relative humidity, %	47.7	8.7	36.9	41.6	44.4	51.3	71.7	9.7

SD, standard deviation; IQR, interquartile range; O_3_, ozone; PM_2.5_, particulate matter ≤ 2.5 μm in diameter; NO_2_, nitrogen dioxide.

## Data Availability

The data set will be available from the corresponding author, upon reasonable request.

## Supplementary Materials

The following supporting information can be downloaded at: https://www.mdpi.com/article/10.3390/toxics13030196/s1, Table S1: Association of various indoor and outdoor ozone concentrations with daily nasal and respiratory symptoms. (a) Maximum 1-h value; (b) Maximum 8-h average; (c) 24-h average, after additional adjustment for nitrogen dioxide; Table S2: Association of various indoor and outdoor ozone concentrations with daily nasal and respiratory symptoms. (a) Maximum 1-h value; (b) Maximum 8-h average; (c) 24-h average, among the restricted subjects without missing record during the study period; Table S3: Comparison of characteristics between subjects who measured personal ozone exposure and those without the measurement.

## Abbreviations

The following abbreviations are used in this manuscript:

Ox	photochemical oxidant
US EPA	United States Environmental Protection Agency
VOCs	volatile organic compounds
O_3_	ozone
WHO	World Health Organization
IFN-γ	interferon-gamma
PM_2.5_	particulate matter ≤ 2.5 μm in diameter
NO_2_	nitrogen dioxide
OR	odds ratio
CI	confidence intervals
IQR	interquartile range
SD	standard deviation
