# Supplementary material for: Short-Term Effects of Exposure to Atmospheric Ozone on the Nasal and Respiratory Symptoms in Adolescents
Source: Toxics. 2025 Mar 9;13(3):196. doi: 10.3390/toxics13030196 (PMC11945642; doi:10.3390/toxics13030196)
Supplement: Supplementary file 1 [file toxics-13-00196-s001.zip › toxics-3503970-supplementary.pdf]

SUPPLEMENTARY MATERIALS

Short-term effects of exposure to atmospheric ozone on the nasal and respiratory symptoms in adolescents

Yoshiko Yoda, Takeshi Ito, Junko Wakamatsu, Tomonari Masuzaki and Masayuki Shima

Table S1. Association of various indoor and outdoor ozone concentrations with daily nasal and respiratory symptoms. (a) Maximum 1-h value; (b) Maximum 8-h average; (c) 24-h average, after additional adjustment for nitrogen dioxide.

Table S2. Association of various indoor and outdoor ozone concentrations with daily nasal and respiratory symptoms. (a) Maximum 1-h value; (b) Maximum 8-h average; (c) 24-h average, among the restricted subjects without missing record during the study period.

Table S3. Comparison of characteristics between subjects who measured personal ozone exposure and those without the measurement.

Table S1. Association of various indoor and outdoor ozone concentrations with daily nasal and respiratory symptoms. (a) Maximum 1-h value; (b) Maximum 8-h average; (c) 24-h average, after additional adjustment for nitrogen dioxide.

	Indoor			Outdoor		
	OR	95%CI	*p* values	OR	95%CI	*p* values
Maximum 1-hour value						
Sneeze	1.01	(0.99, 1.03)	0.363	1.00	(0.99, 1.01)	0.568
Runny nose	1.00	(0.98, 1.03)	0.841	1.02	(1.00, 1.03)	0.015
Nasal congestion	1.02	(1.00, 1.05)	0.044	1.01	(0.99, 1.02)	0.290
Cough	1.01	(1.00, 1.03)	0.077	1.01	(1.00, 1.02)	0.094
Dyspnea	1.00	(0.99, 1.02)	0.597	1.00	(0.99, 1.01)	0.599
Maximum 8-hour average						
Sneeze	1.01	(0.99, 1.03)	0.325	1.02	(1.00, 1.05)	0.097
Runny nose	1.02	(1.00, 1.04)	0.081	1.01	(0.98, 1.04)	0.624
Nasal congestion	1.00	(0.98, 1.02)	0.793	1.01	(0.98, 1.04)	0.560
Cough	1.00	(0.98, 1.01)	0.772	1.00	(0.98, 1.02)	0.856
Dyspnea	1.00	(0.99, 1.02)	0.808	1.01	(0.99, 1.03)	0.405
24-hour average						
Sneeze	1.01	(0.99, 1.03)	0.432	1.01	(0.99, 1.03)	0.308
Runny nose	1.00	(0.97, 1.02)	0.820	1.01	(0.98, 1.03)	0.642
Nasal congestion	1.01	(0.99, 1.03)	0.559	1.00	(0.98, 1.02)	0.956
Cough	1.01	(0.99, 1.02)	0.318	1.01	(0.99, 1.03)	0.232
Dyspnea	1.00	(0.99, 1.02)	0.837	1.00	(0.98, 1.02)	0.829

Table S2. Association of various indoor and outdoor ozone concentrations with daily nasal and respiratory symptoms. (a) Maximum 1-h value; (b) Maximum 8-h average; (c) 24-h average, among the restricted subjects without missing record during the study period.

	Indoor			Outdoor		
	OR	95%CI	*p* values	OR	95%CI	*p* values
Maximum 1-hour value						
Sneeze	1.01	(0.99, 1.03)	0.209	1.00	(0.99, 1.01)	0.842
Runny nose	1.00	(0.98, 1.02)	0.909	1.02	(1.00, 1.03)	0.023
Nasal congestion	1.02	(1.00, 1.04)	0.026	1.01	(1.00, 1.02)	0.258
Cough	1.01	(1.00, 1.03)	0.041	1.01	(1.00, 1.02)	0.020
Dyspnea	1.01	(0.99, 1.02)	0.410	1.00	(0.99, 1.01)	0.575
Maximum 8-hour average						
Sneeze	1.01	(0.99, 1.03)	0.430	1.01	(0.99, 1.04)	0.325
Runny nose	1.02	(1.00, 1.04)	0.055	1.01	(0.98, 1.04)	0.575
Nasal congestion	1.00	(0.98, 1.02)	0.924	1.01	(0.98, 1.04)	0.560
Cough	1.00	(0.99, 1.01)	0.966	1.00	(0.98, 1.02)	0.909
Dyspnea	1.00	(0.99, 1.02)	0.839	1.01	(0.99, 1.03)	0.438
24-hour average						
Sneeze	1.01	(0.99, 1.03)	0.378	1.02	(1.00, 1.04)	0.129
Runny nose	1.00	(0.97, 1.02)	0.685	1.01	(0.98, 1.03)	)0.532
Nasal congestion	1.01	(0.99, 1.03)	0.401	1.00	(0.98, 1.02)	0.771
Cough	1.01	(1.00, 1.02)	0.217	1.01	(1.00, 1.03)	0.062
Dyspnea	1.00	(0.99, 1.02)	0.677	1.00	(0.99, 1.02)	0.614

Table S3. Comparison of characteristics between subjects who measured personal ozone exposure and those without the measurement.

	Subjects who measured personal O_3 exposure (n = 23)	Subjects who did not measure personal O_3 exposure (n =13)	p value
Age (years), mean (SD)	16.1 (0.3)	16.1 (0.3)	0.709
Male, n (%)	13 (56.5)	11 (68.8)	0.440
History of pollinosis and/or allergic rhinitis, n (%)	13 (56.5)	12 (75.0)	0.237
Response to questionnaire on daily symptoms	n = 656	n = 461	
Sneeze (%)	7.4	7.8	0.804
Runny nose (%)	6.7	8.8	0.207
Nasal congestion (%)	6.9	8.7	0.288

O_3, ozone; SD, standard deviation
